# Human Genetic Diseases

**DOI:** 10.1155/2015/315216

**Published:** 2015-05-20

**Authors:** Hao Deng, Peter Riederer, Han-Xiang Deng, Weidong Le, Wei Xiong, Yi Guo

**Affiliations:** ^1^Center for Experimental Medicine and Department of Neurology, The Third Xiangya Hospital, Central South University, Changsha 410013, China; ^2^Clinic and Policlinic for Psychiatry, Psychosomatics and Psychotherapy, University of Wuerzburg, 97080 Wurzburg, Germany; ^3^Division of Neuromuscular Medicine, Davee Department of Neurology and Clinical Neurosciences, Northwestern University Feinberg School of Medicine, Chicago, IL 60208, USA; ^4^Center for Translational Research of Neurology Disease, The 1st Affiliated Hospital, Dalian Medical University, Dalian 11600, China; ^5^Key Laboratory of Carcinogenesis of Ministry of Health and Key Laboratory of Carcinogenesis and Cancer Invasion of Ministry of Education, Cancer Research Institute, Central South University, Changsha 410013, China; ^6^Center for Experimental Medicine, The Third Xiangya Hospital, Central South University, Changsha 410013, China; ^7^Department of Medical Information, Xiangya School of Medicine, Central South University, Changsha 410013, China

There is no question that the rapid advance in genetic technology is changing our viewpoint on medical practice, which is dramatically improving the diagnosis, prognosis, and therapy of human genetic disease. In particular, the next-generation sequencing (NGS) technologies, such as exome sequencing and whole-genome sequencing, and gene editing technology have been applied to several areas, such as genomes, transcriptomes, and epigenomes, and have transformed the genetic research of human diseases. As a powerful and cost-effective discovery and diagnostic tool, exome sequencing was widely used in detecting disease-associated variants underlying genetic disease and developed genetic research such as personalized medicine and personal genomics.

Through rigorous peer review, this special issue includes high-quality papers. We provide a general description as follows.

In the paper, “A Novel* COL4A5* Mutation Identified in a Chinese Han Family Using Exome Sequencing,” X. Xiu et al. have explored the disease-related gene in a four-generation Chinese Han pedigree of Alport syndrome. Their results showed that a novel deletion* COL4A5* mutation, c.499delC (p.Pro167Gln*fs* ^∗^36), may be responsible for AS in this family. Also, their works indicate that exome sequencing is a fast, sensitive, and relatively low-cost method to identify disease-associated mutation(s).

By using NGS, Q. Zhou et al. intended to study the possible association of certain genes with X-linked retinitis pigmentosa (RP) in a Chinese family. They discovered a novel c.C1555T (p.R519T) mutation in the* CACNA1F* gene on X chromosome, which showed perfect cosegregation with the disease in the family. The identification may have significant contribution for the RP diagnosis, genetic counseling, and clinical management.

C. Wang and Q. Tian determined the long-term quality of life (QOL) in Chinese patients with disorders of sex development (DSD). Their works suggest that, compared with the Chinese urban population, the QOL score of DSD patients in China was not significantly lower. With proper treatment, including the follow-up and psychological support, the QOL of DSD patients cannot be significantly reduced, and more attention should be paid to the potential psychological and sexual problems.

A. P. Grillo et al. investigated the association of nine single nucleotide polymorphisms (SNPs) located within the* DFNB1* locus with the occurrence of autosomal recessive nonsyndromic hearing loss (ARNSHL). Their works showed that there were statistically significant differences between patients and controls, and the SNPs presented in the* GJB2* and* GJB6* genes may have an influence on ARNSHL in humans.

R. S. Honjo et al. intended to report the clinical findings of 55 Brazilian Williams-Beuren syndrome (WBS) patients confirmed by Multiplex Ligation-Dependent Probe Amplification (MLPA). Their results indicate that MLPA was a promising method in the diagnostic investigation of WBS and was effective in detecting the microdeletion.

A. Gordon-Shaag et al. summarized the current research development in keratoconus (KC) epidemiology and genetic etiology. Risk factors, including environmental, socioeconomic, and familial factors, were also discussed. The detailed molecular mechanism will significantly advance our understanding of KC and promote the development of potential therapies.

G. N. Cerbino et al. reviewed the molecular heterogeneity of Acute Intermittent Porphyria (AIP) in Argentinean patients. Thirty-five different mutations were identified in the* HMBS* gene, and a founder effect (p.G111R) was found. Their works also indicated the importance of molecular techniques as the most appropriate tools for detecting and identifying specific mutations in carriers of affected families.

R. Farhat et al. intended to explain the variable phenotypes of* CFTR* c.3909C>G mutation in Cystic Fibrosis patients in the Lebanese. They identified the association between the* CFTR* c.3909C>G and complex allele c.[744-33GATT(6); 869+11C>T]. Splicing studies revealed no impact of the c.3909C>G mutation on splicing, whereas the associated complex allele induces minor exon 7 skipping.

With the development of NGS, it has been used for uncovering the genes underlying unsolved Mendelian disorders, explaining the heritability of complex and health-related trains, and even setting the stage for applying to facilitate clinical diagnosis and personalized disease-risk profiling. New mutations, if validated, contribute to find specific and selective biomarkers for certain diseases, which are important prerequisites for early diagnosis and treatment. Few diseases could be due to a single factor, and most common diseases are multifactorial. Exploring the interaction of multiple factors, including modificator genes and environmental and geographical factors, may help us to better understand the genetics mechanism of human disease.

This special issue is intended to develop and expand the association between human disease and genetics. By soliciting paper, we hope this special issue will help stimulate the understanding of molecular pathology underlying human genetics diseases and provide new insight in diagnosis, therapy, and genetic counseling of human genetic disease.

